# Monometallic Cerium Layered Double Hydroxide Supported Pd-Ni Nanoparticles as High Performance Catalysts for Lignin Hydrogenolysis

**DOI:** 10.3390/ma13030691

**Published:** 2020-02-04

**Authors:** Tibo De Saegher, Jeroen Lauwaert, Jorku Hanssen, Els Bruneel, Matthias Van Zele, Kevin Van Geem, Klaartje De Buysser, An Verberckmoes

**Affiliations:** 1 Industrial Catalysis and Adsorption Technology (INCAT), Department of Materials, Textiles and Chemical Engineering (MaTCh), Faculty of Engineering and Architecture, Ghent University, Valentin Vaerwyckweg 1, 9000 Ghent, Belgium; Tibo.DeSaegher@UGent.be (T.D.S.); Jeroen.Lauwaert@UGent.be (J.L.); Jorku.Hanssen@UGent.be (J.H.); 2 Sol-Gel Centre for Research on Inorganic Powders and Thin Films Synthesis (SCRiPTS), Department of Chemistry, Faculty of Sciences, Ghent University, Krijgslaan 281 S3, 9000 Ghent, Belgium; Els.Bruneel@UGent.be (E.B.); Matthias.VanZele@UGent.be (M.V.Z.); Klaartje.DeBuysser@UGent.be (K.D.B.); 3 Laboratory for Chemical Technology (LCT), Department of Materials, Textiles and Chemical Engineering (MaTCh), Faculty of Engineering and Architecture, Ghent University, Technologiepark 125, 9052 Ghent, Belgium; Kevin.VanGeem@UGent.be

**Keywords:** layered double hydroxide, PdNi nanoparticles, lignin hydrogenolysis, cerium

## Abstract

Monometallic cerium layered double hydroxides (Ce-LDH) supports were successfully synthesized by a homogeneous alkalization route driven by hexamethylenetetramine (HMT). The formation of the Ce-LDH was confirmed and its structural and compositional properties studied by XRD, SEM, XPS, iodometric analyses and TGA. HT-XRD, N_2_-sorption and XRF analyses revealed that by increasing the calcination temperature from 200 to 800 °C, the Ce-LDH material transforms to ceria (CeO_2_) in four distinct phases, i.e., the loss of intramolecular water, dehydroxylation, removal of nitrate groups and removal of sulfate groups. When loaded with 2.5 wt% palladium (Pd) and 2.5 wt% nickel (Ni) and calcined at 500 °C, the PdNi-Ce-LDH-derived catalysts strongly outperform the PdNi-CeO_2_ benchmark catalyst in terms of conversion as well as selectivity for the hydrogenolysis of benzyl phenyl ether (BPE), a model compound for the α-O-4 ether linkage in lignin. The PdNi-Ce-LDH catalysts showed full selectivity towards phenol and toluene while the PdNi-CeO_2_ catalysts showed additional oxidation of toluene to benzoic acid. The highest BPE conversion was observed with the PdNi-Ce-LDH catalyst calcined at 600 °C, which could be related to an optimum in morphological and compositional characteristics of the support.

## 1. Introduction

Evolutions towards a circular sustainable economy, including the replacement of fossil resources by renewable alternatives are expected to become increasingly important for, a.o., the abatement of climate change. Therefore, over the past decade, many biorefinery processes have emerged, which in some cases can replace a part of the petrochemical industry, although today they lack competitiveness [1]. Lignocellulose (e.g., hard and soft wood, wheat straw, switchgrass, etc.) is the most abundant and cheapest inedible biomass source and, hence, has been identified as the most scalable and economically viable bio-source for sustainable production of both bio-fuels and high value chemicals [2]. Lignocellulosic biomass is mainly composed of three biopolymers, i.e., cellulose, hemicellulose and lignin.

Since the emergence of the lignocellulosic based biorefinery concept, research has mainly focused on converting cellulose and hemicellulose into consumables, i.e., fuels, chemicals, polymers, medicines, etc. [3,4]. So far, lignin has received less attention because of its chemical heterogeneity, complexity and low reactivity and is, currently, not optimally exploited. Traditionally, most large-scale industrial processes that use plant polysaccharides have burned lignin as a low value fuel to generate the power needed to productively transform the biomass [5,6,7]. However, as lignin is the largest sustainable aromatic feedstock, it constitutes a worthy resource for materials’ applications or for the production of renewable aromatics. 

**Figure 1:**
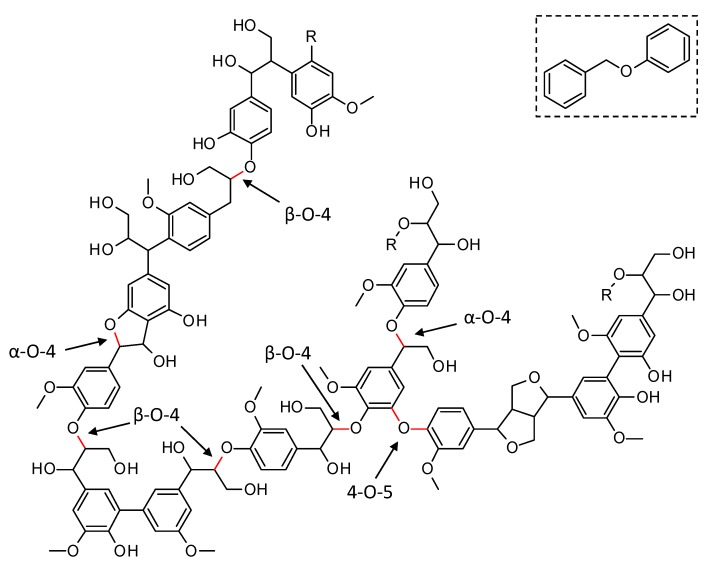
Representation of a native lignin structure with indication of the common C_aryl_-O ether linkages (in red). Benzyl phenyl ether (top right) is the model compound for the α-O-4 ether linkage in this work.

Lignin has a complex molecular structure that mainly consists of phenylpropanoid units, i.e., coumaryl, coniferyl and sinapyl alcohols [8]. Other building blocks such as ferulate, p-coumarate, hydroxycinnamaldehyde, etc. are typically incorporated to a lesser extent. Several different types of linkages connect the monomers via ether bonds and/or carbon-carbon bonds (C–C). The former (e.g., β-O-4, α-O-4, 4-O-5, in Figure 1), typically, predominate in native lignin and are significantly weaker than the C–C bonds, making the cleavage of the C_aryl_–O bonds an interesting approach for lignin depolymerization. Although most current technical lignins show a significantly lowered amount of ether bonds, in favor of C–C linkages, due to the severe conditions applied during lignin extraction, it is expected that the emerging biorefinery concept will shift towards milder lignin isolation techniques, which prevent the non-selective ether bond scission [8,9,10,11]. Model compounds, resembling native ether linkages in lignin have been used extensively in literature to assess the performance of catalysts in the depolymerization and to get in-depth insights into the occurring reaction pathways [12,13,14,15,16,17]. In this work, the performance of synthesized catalysts for the reductive depolymerization of lignin is assessed through benzyl phenyl ether (BPE), a model compound resembling the α-O-4 ether linkage and commonly used throughout the literature (see Figure 1, top right) [15,16,18].

Several depolymerization strategies have been developed, e.g., reductive, oxidative, base and acid catalyzed depolymerizations, each of them following different reaction mechanisms and, hence, yielding very different products. Amongst these strategies, the reductive depolymerization has been identified as a reaction exhibiting both high yield and selectivity towards monomeric, phenolic species [4,5,19]. During the reductive depolymerization, also referred to as hydrogenolysis, the ether bonds in lignin are cleaved in the presence of a redox catalyst and a reducing agent [4,20]. When hydrogen is used as a reducing agent, the process is typically called hydro-processing while the term liquid-phase reforming is used when the hydrogen is derived from a hydrogen donating component, e.g., the solvent. Several supported noble (e.g., Pd, Pt, Rh or Ru) and base (e.g., Ni or Cu) metals have been studied as potential catalysts for this reaction. Moreover, next to the heterogeneous catalyst, the addition of homogeneous co-catalysts such as Lewis acids (CrCl_3_) or bases (NaOH or KOH) has been used in order to enhance the monomer yields [4]. Therefore, it might be hypothesized that multifunctional materials containing both redox and alkaline properties will have high potential in this application. 

The first essential property of a catalyst for the reductive depolymerization of lignin is the incorporation of redox active metals. As aforementioned, several supported noble metals (Pd, Pt, Rh, Ru, etc.) and base metals (Ni or Cu) have been studied [4]. In recent years, the usage of nanoparticle catalysts, both within this specific application and within the broad field of catalysis itself, has attracted a lot of attention due to their high activity as a result of their large surface area to volume ratio. Moreover, combining two metals in nanoparticle catalysts can lead to performances far exceeding those of the individual metals, due to the synergistic interactions between the metals. More specifically, for the selective cleavage of aromatic ether linkages, such as the β-O-4 and α-O-4 links in lignin, the combination of palladium and nickel has shown high potential [14]. As palladium exhibits a high activity in the cleavage of dimeric aromatic ether linkages but poor selectivity towards aromatic products (due to hydrogenation of the aromatic rings) and nickel shows high selectivity but low activity, combining them in nanoparticles has resulted in catalysts with both high activity and selectivity. Therefore, within this work, palladium nickel nanoparticles have been synthesized and tested.

In addition to metal nanoparticles, a suitable support, which further promotes their performance through synergistic interactions, is crucial for the materials’ catalytic performance. Traditionally, metal oxides have been considered in the context of the reductive lignin depolymerization, as they provide key characteristics for efficient and effective cleavage of ether bonds [4,21]. Firstly, most metal oxides contain both Lewis acid sites and Brønsted or Lewis basic sites that can attract hydrides (H:) or protons (H^+^) from alcohols, respectively, lowering the activation energy of reaction steps involving a hydrogen transfer. Secondly, metal oxides seem to stabilize the reactive intermediates, which are formed during reductive lignin depolymerization, e.g., through their alkylation with solvent molecules (e.g., ethanol or methanol). As a result, unwanted repolymerization can be avoided [21]. Alumina (Al_2_O_3_) and silica (SiO_2_), which show many of the aforementioned beneficial properties, have been examined extensively for the reductive depolymerization of lignin [4]. However, ceria (CeO_2_), or in general ceria containing supports, have shown similar interesting properties within the broad field of catalysis [22]. First, ceria is known for affecting the dispersion of supported metals, increasing the thermal stability of the supporting material, promoting noble metal reduction and oxidation and, most notably, having a unique redox capability, shifting between Ce^3+^ and Ce^4+^ under net reductive and oxidative conditions respectively [22]. Therefore, they are used as catalyst supports for dry reforming of toluene and methanol steam reforming in industry [23,24]. Moreover, specifically beneficial for the hydrogenolysis of lignin, ceria is known to promote the water gas shift and steam reforming reactions, which are closely related to the ability of the support to generate hydrogen in situ. Additionally, ceria has the ability to store and release hydrogen [21,22]. AuPd/CeO_2_ catalysts have already been tested in reductive depolymerization of lignin and model compounds and yielded promising results [16].

However, research towards the development of novel and innovative catalytic systems continuously leads to new insights and different approaches in catalyst design and application. As a consequence, next to metal oxides, layered double hydroxides are another emerging class of porous materials showing great potential as nanoparticle supports [25]. Layered double hydroxides (LDHs) or hydrotalcite-like components comprise a class of highly versatile anionic clay materials consisting of positively charged brucite-like layers, which have intercalated water and exchangeable charge-compensating anions, e.g., OH^−^, SO_4_^2−^, CO_3_^2−^ or NO_3_^−^ [2]. The lamellar layers are composed of octahedrons formed by sharing their edges, with metal cations in the center and six hydroxide ions at the vertices [2,26,27]. As a result, these materials are characterized by a uniform dispersion of the metal cations throughout the layers and act as solid bases. Moreover, the basicity is tunable in both nature (Brønsted and Lewis type basic sites) and strength (from weak–middle to strong basic sites) [28,29]. Additionally, the cations of brucite-like layers are also tunable and the anions are exchangeable, which provides a wide versatility in LDH composition [2,30]. Another interesting feature is the memory effect by which the oxide obtained by the thermal decomposition of the LDH can be reconverted into the original LDH structure upon contact with water or aqueous solutions containing certain anions [31]. As a result of their extraordinary properties, LDH materials are widely used in a large variety of applications, e.g., catalysis, drug delivery systems, adsorbents, among many others [32]. LDHs can be synthesized directly via co-precipitation [33], the sol-gel method [34,35], the self-combustion method [36], or the homogeneous alkalization route [37,38], or indirectly by the modification of pre-synthesized LDHs via anion exchange methods aiming at the substitution of the interlayer anions by other anions or even medicines (producing drug delivery systems), or reconstruction and rehydration methods [39]. It should be noted that the direct homogeneous alkalization route is especially suitable for synthesis of highly crystalline LDHs [37,38].

**Figure 2:**
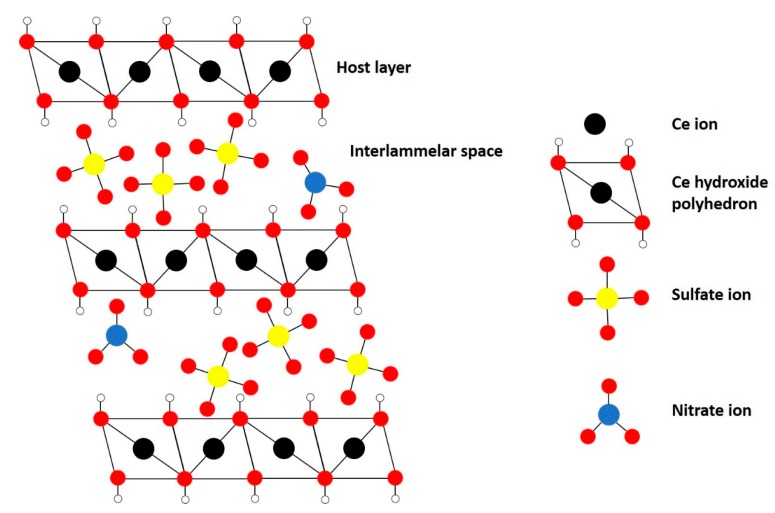
Structure of monometallic Ce-LDH.

Traditional LDHs, e.g., hydrotalcite (HT), contain two different metals with different oxidation states (often 2+ and 3+) in their structure to introduce a net positive charge in the host layer that is compensated by the interlayer anions. However, more recently, monometallic layered double hydroxides, i.e., incorporating one metal with multiple oxidation states, have been successfully synthesized, e.g., monometallic cobalt LDH (Co^2+^/Co^3+^), layered rare-earth hydroxides (LREH) and monometallic cerium LDH [29,38,40,41,42,43]. The latter was synthesized successfully by Ye et al., to the best of our knowledge, for the first time through a novel homogeneous alkalization route [38]. The material consists of a layered structure of Ce-hydroxy polyhedra (Ce^3+^ and Ce^4+^) with nitrate and sulfate ions in the interlayers as schematically illustrated in Figure 2. The Ce-LDH proved to be a successful catalyst for photocatalytic reduction of CO_2_ due to the ability of cerium to cyclically switch between Ce^4+^ and Ce^3+^.

The monometallic Ce-LDH combines the aforementioned beneficial catalytic properties of ceria-containing supports with the presence of surface hydroxyl groups and intercalated basic anions. Moreover, as the presence of both Brønsted and Lewis basic species has been reported to boost the hydrogenolysis of lignin, the Ce-LDH material is expected to constitute an interesting supporting material for this application [44]. Additionally, LDHs offer a combined steric–electrostatic stabilization, which combined with the beneficial effects of ceria containing materials on the dispersion of metals, can lead to small, well dispersed and stabilized metal nanoparticles [22,25]. However, to the best of our knowledge, the application of monometallic Ce-LDH materials as nanoparticle support, let alone for hydrogenolysis of lignin specifically, has not been reported in literature. Therefore, within this work, a monometallic cerium double layered hydroxide has been synthesized through hexamethylenetetramine (HMT)-driven homogeneous alkalization route [38]. The structural and compositional properties were evaluated through X-ray diffraction (XRD), scanning electron microscopy (SEM), X-ray photoelectron spectroscopy (XPS), iodometric titration and thermogravimetric analysis (TGA). As calcination of LDH structures at different temperatures leads to new materials with different compositional and morphological properties, which ultimately also affects their performance as catalyst support materials, the effect of the calcination temperature on these properties was evaluated through high temperature XRD (HT-XRD), nitrogen sorption and X-ray fluorescence spectroscopy (XRF) [38,41]. Finally, the synthesized Ce-LDH materials, calcined at different temperatures, were loaded with 2.5 wt% palladium (Pd) and 2.5 wt% nickel (Ni), calcined at 500 °C and their performance in the reductive depolymerization of lignin was evaluated through the reductive cleavage of the α-O-4 ether linkage in BPE. 

## 2. Materials and Methods

### 2.1. Synthesis Procedures 

#### 2.1.1. Monometallic Cerium LDHs

The monometallic cerium LDHs are synthesized via an optimized homogeneous alkalization route as described by Ye et al [38]. In case of the benchmark synthesis scale, 0.434 g cerium(III)nitrate hexahydrate (Ce(NO_3_)_3_ 6H_2_O, Sigma-Aldrich, 99% purity), 10 mL 0.5M hexamethylenetetramine (C_6_H_12_N_4_, Chimica, 98.5% purity), 1.2 mL 0.5 M ammonium persulfate ((NH_4_)_2_S_2_O_8_, Sigma-Aldrich, 98% purity) and 0.760 g sodium chloride (NaCl, Acros Organics, 99.5% purity) are dissolved in 200 mL of distilled water in a round-bottom flask, which is subsequently closed off with a septum and purged with argon gas to create an inert atmosphere. Subsequently, the mixture is refluxed for 24 h at a temperature of 110 °C under stirring at 400 rpm. Finally, the obtained precipitate is removed by centrifugation, washed three times with distilled water and once with ethanol (Chem-Lab Analytical, 100% abs.) and dried for 48 h in air at room temperature. This material is denoted as Ce-LDH. 

Additionally, in order to obtain a single larger homogeneous batch of material (±2.5 g) for subsequent various heat/calcination treatments, the synthesis recipe as described above has also been scaled up by increasing the amount of reactants by a factor of four and dissolving them in 500 mL of distilled water. Hence, the concentrations within this synthesis were slightly higher than in the benchmark synthesis, i.e., by a factor of 1.6. Afterwards, the scaled up Ce-LDH supports are calcined under air by heating at a ramp of 2 °C/min to a specific temperature and maintaining that temperature for 6 hours. The effect of this calcination temperature on the support characteristics has been analyzed for the following values: 200 °C, 400 °C, 600 °C and 800 °C. Throughout this work, the resulting support materials are denoted as Ce-LDH-200, Ce-LDH-400, Ce-LDH-600 and Ce-LDH-800, respectively. 

#### 2.1.2. Cerium Oxide Support

About 1 g of CeO_2_ has been prepared via a hydrothermal procedure, inspired by that described by Gao et al [16]. Firstly, 2.522 g cerium(III)nitrate hexahydrate (Ce(NO_3_)_3_ 6H_2_O, Sigma-Aldrich, 99% purity) is added to 90 mL of a 6 M sodium hydroxide solution (NaOH, Sigma-Aldrich, 99% purity). Subsequently, the mixture is placed in an ultrasonic bath for 10min. The mixture is then hydrothermally treated in an autoclave at 100 °C for 24 h under autogenous pressure. Afterwards, the mixture is centrifuged, washed with water and dried at 80 °C for 12 h under air. Finally, even though it is very probable that the CeO_2_ support is already partially crystallized after the hydrothermal synthesis, the material is calcined at 400 °C for 4 h [45].

#### 2.1.3. Palladium Nickel Catalysts 

The calcined supports are loaded with 2.5 wt% Ni and 2.5 wt% Pd via incipient wetness impregnation. This technique was chosen because the involved capillary forces result in improved particle dispersions as compared to classical wet impregnation where the impregnation is governed by diffusion, to limit deposition at the outside surface of the support and to minimize waste [46]. To perform the impregnation, 0.3 g of support is analytically weighed. Subsequently, the solution containing the metal precursors palladium(II)nitrate dihydrate (Pd(NO_3_)_2_ 2H_2_O, Acros Organics, 39% Pd) and nickel(II)nitrate hexahydrate (Ni(NO_3_)_2_ 6H_2_O, UWB, 99% purity) is prepared by dissolving 0.0179 g and 0.0355 g of Pd(NO_3_)_2_ 2H_2_O and Ni(NO_3_)_2_ 6H_2_O, respectively, in 0.5 mL of distilled water. This solution is added to the support under mechanical mixing with a spatula to ensure a uniform loading of the support. Afterwards, the impregnated support is dried overnight at 105 °C under air to allow the water to evaporate. Finally, the dried catalyst is calcined at 500 °C (heating at 10 °C/min) for 4 h under air, converting the metal nitrates into their respective metal oxides [47,48]. The catalysts are denoted as PdNi-Ce-LDH-xxx where ‘xxx’ indicates the calcination temperature of the Ce-LDH support, prior to metal impregnation, as described in Section 2.1.1.

### 2.2. Chemical Analysis and Characterization

#### 2.2.1. X-Ray Diffraction

The crystal phases of the catalysts were characterized by X-ray diffraction (XRD) using a Thermo Scientific ARL X’TRA X-ray powder diffractometer equipped with a copper X-ray tube (Kα λ = 0.15406 nm). The scattering intensities were measured with a step width of 0.02° and at a rate of 0.8°/min. 

The Ce-LDH support and PdNi-Ce-LDH catalysts were measured over an angular range of 5°< 2θ < 60° and 10° < 2θ < 40° respectively. The detector, a Peltier DetectorTM, is a cooled lithium doped silicium solid state detector. The Rietveld method for whole-powder pattern fitting was used and the Topas Academic V4.1 software (Brisbane, Australia) was used for Rietveld refinement of the scaled-up Ce-LDH XRD pattern. The refined parameters were the measurement specific or sample displacement error, a cosine Chebyshev function of 10 polynomial terms for background correction, phase specific scale factors, unit cell parameters and Lorentzian peak shape broadening parameters. Additionally, HT (high temperature); X-ray diffraction experiments were performed using a D8 Discover (Bruker Corporation, Billerica, MA, USA) with a CuKα1 source. A 2θ scan was made from 20° to 60° in steps of 0.05°, 1 s scanning time per step. During this analysis, the sample was heated up to 1000 °C at 10 °C/min.

#### 2.2.2. Scanning Electron Microscopy and Energy-Dispersive X-Ray Spectroscopy

The morphologies of the Ce-LDH precipitate and different catalysts, loaded with Pd and Ni and calcined at 500 °C, are examined via scanning electron microscopy (SEM) analysis. The SEM images are recorded on an FEI Quanta FEG 200 (Fei, Hilboro, OR, USA). Additionally, SEM-energy dispersive X-ray spectroscopy (SEM-EDX) measurements and mappings for Pd and Ni were recorded using the FEI Quanta FEG 200, fitted with an EDAX Genesis 4000 EX system, and a Jeol JSM-7600F FEG SEM (Jeol, Tokyo, Japan), equipped with Oxford X-Max EDX spectrometer, respectively.

#### 2.2.3. Thermogravimetric Analysis

A thermogravimetric analysis (TGA) was performed to gain insight into the thermal degradation and the structural formula of the synthesized materials. A STA 449 F3 Jupiter device and Proteus Thermal Analysis program from NETZSCH (Selb, Germany) are used. ±10 mg Ce-LDH is heated up to 1000 °C at a rate of 10°/min under air.

#### 2.2.4. Fourier-Transform Infrared Spectroscopy

Fourier-transform infrared spectroscopy (FT-IR) measurements were performed on a Bruker Equinox 55 FT-IR spectrometer (Billerica, MA, USA) to gain insight into the composition of the Ce-LDH derived materials. The dried samples were placed on a potassium bromide (KBr) pellet and measured under vacuum at room temperature.

#### 2.2.5. Nitrogen Adsorption

The specific surface area of the supports was determined by means of N_2_ adsorption and desorption at −196 °C in a Micromeritics Tristar 3000 (Norcross, GA, USA). Prior to the analysis the samples were degassed for 2 h at 120 °C and vacuum. The specific surface area was determined by fitting the adsorption and desorption curves to the BET-isotherm.

#### 2.2.6. X-Ray Fluorescence

To determine the sulfur content of the Ce-LDH, as a function of the calcination temperature, X-ray fluorescence (XRF) analysis was performed. The XRF-spectra of the powder samples were recorded under air on a Rigaku NeXCG (Tokyo, Japan) where the primary X-rays are delivered by a copper X-ray tube. The detection is performed by a multi-channel analyzer.

#### 2.2.7. X-Ray Photoelectron Spectroscopy

The presence of both Ce^3+^ and Ce^4+^ in the Ce-LDH materials is confirmed through X-ray photoelectron spectroscopy (XPS) (SSI, Mountain View, CA, USA) and the Ce^4+^ fraction m ($n_{{\mathrm{Ce}}^{4+}}/n_{{\mathrm{Ce}}^{4+}}+n_{{\mathrm{Ce}}^{3+}}$) is determined. The analyses were performed in a Surface Science Instruments S-Probe XPS spectrometer with monochromatic Al radiation (1486 eV) and controlled with S probe ESCA software version 1.36.04). Adventitious carbon was used for calibration and the flood gun was set to 3 eV.

#### 2.2.8. Iodometric Titration

The Ce^4+^/Ce^3+^ ratios of Ce-LDH and CeO_2_ were also determined through iodometric titration, which quantifies the amount of Ce^4+^ atoms in the material [49,50]. 20 mg of material was dissolved in 20 mL 3M HCl solution and placed in an ultrasonic bath for 2 h at 85 °C. Afterwards 40 mL of a 0.1 M potassium iodide (KI, Chem-Lab, 99.5% purity) solution is added, resulting in the reduction of the present Ce^4+^ to Ce^3+^, according to the following redox reaction:

$${\mathrm{Ce}}^{4+}+I^{-}\to {\mathrm{Ce}}^{3+}+\frac{1}{2}I_{2}$$

The resulting molecular iodine can be titrated with a solution of sodium thiosulfate (Na_2_S_2_O_3_, Chem-Lab, >99.5% purity) of which the concentration ($C_{Na_{2}S_{2}O_{3}}$) is determined by titration with a 0.01 N potassium bromate (KBrO_3_, VW, 100% purity) solution, with starch ((C_6_H_10_O_5_)_n_, VWR, >79.5% purity) as indicator, according to the following redox reaction:

$$I_{2}+2S_{2}O_{3}^{2-}\to 2I^{-}+S_{4}O_{6}^{2-}$$

Based on these results, the fraction of Ce^4+^ in the Ce-LDH (m), a crucial parameter for the characteristics of the material, can be calculated via the following equation:

$$m=n_{{\mathrm{Ce}}^{4+}}/\;\left(n_{{\mathrm{Ce}}^{4+}}+n_{{\mathrm{Ce}}^{3+}}\right)$$

$${\mathrm{with}\;n}_{{\mathrm{Ce}}^{4+}}=V_{{\mathrm{Na}}_{2}S_{2}O_{3}}\times C_{{\mathrm{Na}}_{2}S_{2}O_{3}}$$

### 2.3. Reaction Procedure 

#### 2.3.1. Reactor Set-Up

The catalytic performance of the synthesized materials has been evaluated in an Eco-cat 6-25-SS316 produced by AmAr (Mumbai, India). This setup contains six individual cylindrical reactor vessels of 25 mL, which can withstand pressures up to 100 bar and temperatures up to 200 °C. The reactors can be supplied with pressurized gas, i.e., hydrogen or nitrogen, via a central chamber and are heated to and maintained at the desired temperature, i.e., 100 °C for catalyst pre-reduction or 150 °C for hydrogenolysis of BPE, using an IKA (Staufen, Germany) RCT basic heating plate equipped with a thermocouple, which is placed in a separated thin tube. Uniformization of the reaction mixtures is ensured by means of magnetic stirrers, which are placed in each reactor vessel and are operated at 900 rpm. 

#### 2.3.2. Catalyst Pre-Reduction.

Before the reaction is performed, the catalysts are reduced in situ through mild solvothermal reduction at 100 °C. This method was chosen as the presence of nickel oxide next to noble metals can lead to synergistic effects, e.g., preventing ring hydrogenation otherwise leading to undesired unsaturated products [51]. To each operated reactor vessel, 100 mg of the desired calcined catalyst is added. Subsequently, 10 mL of methanol (Chem-Lab, >99.8% purity) is added. The reactor is sealed, 10 bar of hydrogen gas is applied, the reactor is heated to 100 °C and the reduction is performed at this temperature for 2 h under 900 rpm of stirring. When the reduction time has passed, the reactor is quenched in an ice bath for at least 20 min. This hydrothermal pre-reduction method is sufficiently strong to reduce palladium oxide but it will likely not suffice in reducing nickel oxide [52,53]. However, adding palladium oxide to base metal oxides (e.g., Cu or Ni) leads to greatly increased reducibility, possibly resulting in the reduction of some PdNi phases during in situ reduction or during the subsequent reaction in methanol at 150 °C [54].

#### 2.3.3. Reductive Cleavage of α-O-4 Model Component 

To perform the reaction, a solution of 10 mL consisting of 1500 ppm benzyl phenyl ether (BPE, Alfa Aesar, 97% purity) and 750 ppm biphenyl (Alfa Aesar, 99% purity, internal standard) in methanol (Chem-Lab, >99.8% purity), is added to each reactor, containing the pre-reduced catalyst in 10 mL of methanol, resulting in a total volume of 20 mL. The reactor is sealed and pressurized with 10 bar of hydrogen gas. The reaction is performed for 1 h at a temperature of 150 °C after which the reactor is quenched in an ice bath. The solid catalysts are separated from the reaction mixture via centrifugation (Himac CT 6EL) at 3000 rpm for 5 min. The liquid phase is recovered and analyzed using reversed phase high performance liquid chromatography (RP-HPLC). Every experiment is repeated multiple times yielding results within a standard deviation of approximately 2% from the average value.

#### 2.3.4. Reversed Phase High Performance Liquid Chromatography

RP-HPLC, in combination with UV-Vis spectroscopy, was utilized to study the BPE conversion and the product selectivity. A Perkin Elmer (Waltham, MA, USA) HPLC system was used, consisting of a non-thermostated column, manual injection valve with 20 µL loop and a UV-Vis detector. The analytical column is packed with a C18 stationary phase: Thermo Scientific™ Hypersil™ ODS C18 Octadecyl silica. The silicone surfaces are modified with octadecyl groups (C18). The mobile phase, i.e., 60% methanol/40% water (Vol/Vol), flows at 1 mL/min. 

The UV–Vis detector was set to 220 nm as BPE, phenol and toluene show sufficient absorbance at this wavelength. Quantification of the different components in the reaction mixture was performed by relating their peak surface areas to the amount and peak area of the internal standard (biphenyl) added to the reactor.

## 3. Results and Discussion

### 3.1. Characterization of the Ce-LDH Support

#### 3.1.1. X-Ray Diffraction 

**Figure 3:**
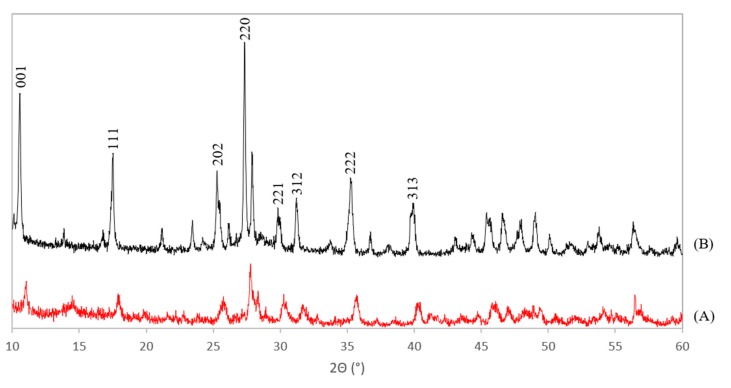
XRD patterns of the benchmark scale Ce-LDH (**A**) and the scaled-up Ce-LDH with Miller indices appointed to the reflections (**B**).

The XRD patterns of the Ce-LDH, prepared through HMT-driven homogeneous alkylation of both the benchmark scale (A) and the scale up (B) are presented in Figure 3. XRD patterns of the Ce-LDH materials synthesized within this work agree well with that of the Ce-LDH as synthesized by Ye et al [38]. Especially the typical reflections at low 2θ values (10° and 17°), for layered structures with an interplanar distance (d-spacing) of 0.838 and 0.419 nm, can be found in both patterns in Figure 3 [38]. Moreover, these reflections also appear in the XRD pattern of rare earth metal layered double hydroxides (LREHs) with intercalated sulfate anions, synthesized by Liang et al [41]. Hence, this confirms the formation of an LDH structure. The cell parameters of the benchmark scale Ce-LDH and scale up Ce-LDH were determined from the obtained XRD patterns and the resulting values are listed in Table 1. The orthorhombic type Bravais unit cell was assumed as this lattice type has also been experimentally confirmed for several LREHs [41]. Comparing the near identical cell parameters for the benchmark scale and scaled-up Ce-LDH further substantiates that the scale-up was successful. These cell parameters were also used to attribute Miller indices to the largest reflections in Figure 3. Both these indices and the calculations to obtain these indices can be found in the supportive information, see Table S1. 

#### 3.1.2. Scanning Electron Microscopy 

**Figure 4:**
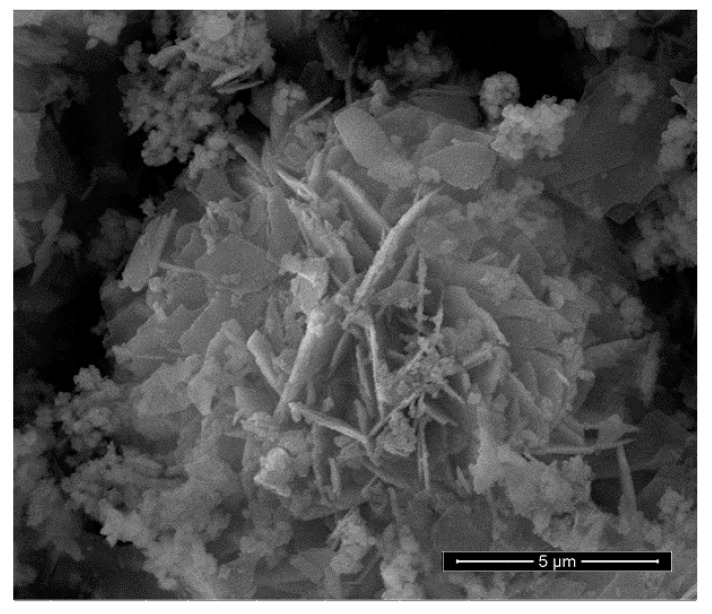
SEM image of the synthesized Ce-LDH, synthesized through HMT-driven homogeneous alkalization.

A SEM image of the benchmark scale Ce-LDH support material is presented in Figure 4. The structure consists of thin plates with a size of 2–4 µm and a thickness of about 100–200 nm. Furthermore, Irregular and smaller particles are also visible, which likely consist of very small plate-like structures with the same composition. Moreover, these small plate-like structures are also visible in the SEM images reported by Ye et al. [38]. The larger plate-like structures are indicative of an LDH structure, be it bimetallic LDHs (e.g., MgAl and CaAl), LREHs or monometallic cobalt layered double hydroxides [35,41,55]. Moreover, Ye et al. also reported plate-like structures of similar sizes for Ce-LDH. Therefore, SEM-imaging also indicates that the Ce-LDH structure is achieved [38].

#### 3.1.3. X-Ray Photoelectron Spectroscopy

**Figure 5:**
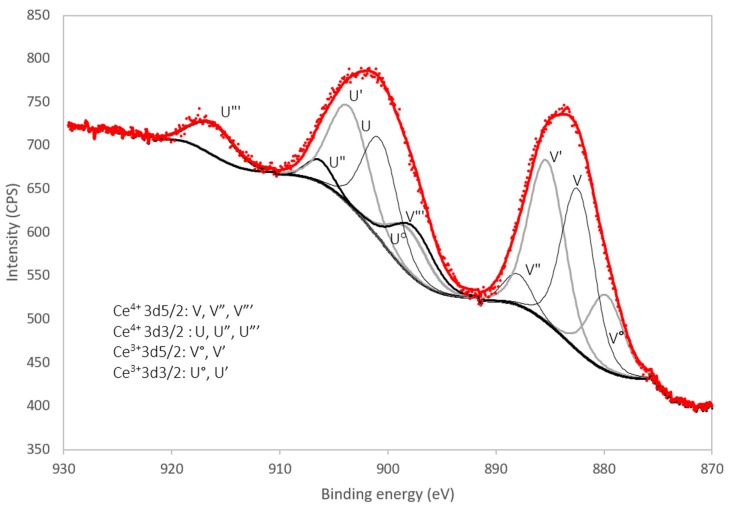
XPS spectrum of the benchmark scale Ce-LDH Ce 3d region (870 eV–930 eV) with peak deconvolution.

The Ce3d region of the XPS spectrum (i.e., between 870 eV and 930 eV) of the benchmark scale Ce-LDH material is presented in Figure 5. The Ce^4+^ 3d spectrum is composed of 6 peaks due to previously described final state effects associated with charge transfer [56,57]. Likewise the Ce^3+^ 3d spectrum is composed out of 4 peaks [58].

Experimental spectra were processed with the CasaXPS (Casa Software Ltd., Teignmouth, UK) software package. Peak deconvolution using 10 mixed Gaussian/Lorentzian curves, Shirley background subtraction and a constraint of the peak position within 0.2 eV of literature values shows a good fit for a 50% Ce^3+^/50% Ce^4+^ composition, which is in agreement with the results of the iodometric titration in Section 3.1.4 and the results reported by Ye et al. [38].

The survey spectrum is presented in Figure S1 (see supportive information) and shows the S2p peak at 168.5 eV, confirming a high oxidation state (+6) of sulfur as in sulfate ions [58,59]. The presence of silicon contamination is observed as the Si2s peak at 153.5 eV [59,60]. No N1s peaks, expected at 407 eV for nitrate groups, could be observed [61]. This absence can be explained by the TGA results reported in Section 3.1.5 as within this analysis, the amount of nitrate anions in the LDH structure was determined to be very small. O1s peaks are observed at 531.2 eV and a minor peak at 527.5 eV. 

#### 3.1.4. Iodometry

The Ce^4+^ contents of both CeO_2_ and the benchmark scale Ce-LDH material were determined through iodometry, see Table 2. The cerium oxide material denoted as CeO_2_, indeed, mainly consists of Ce(IV)O_2_. However, apparently also a small fraction of Ce_2_(III)O_3_ is present in this material, i.e., about 7.6%. Similar amounts of Ce^3+^ in CeO_2_ have been reported in literature and their presence has been attributed to oxygen vacancies at the surface of CeO_2_, resulting in electrons that can reduce the Ce^4+^ to Ce^3+^ [62,63]. In contrast, the Ce^4+^ fraction within the Ce-LDH is significantly lower as compared that of CeO_2_, i.e., about 46.4%, which is in good agreement with the value, determined through XPS analysis in Section 3.1.3. (50%). Moreover, this nearly equimolar ratio between Ce^4+^ and Ce^3+^ was also observed by Ye et al., further confirming the formation of a Ce-LDH structure [38].

#### 3.1.5. Thermogravimetric Analysis

**Figure 6:**
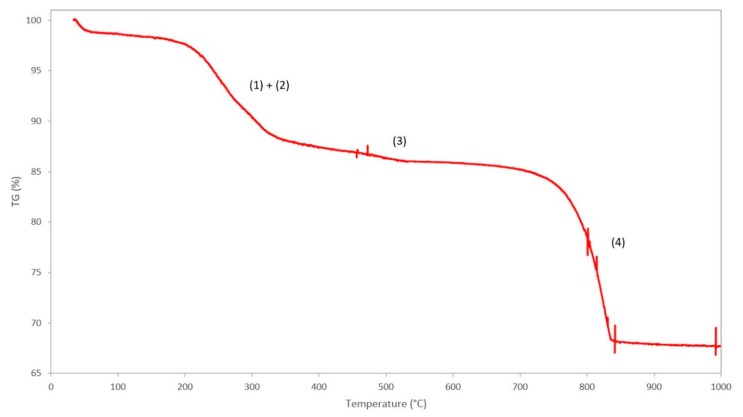
TGA curve of the Ce-LDH and proposed degradation reactions: (1) removal of intramolecular water, (2) dehydroxylation, (3) removal of nitrate groups and (4) removal of sulfate groups.

The TGA curve of the benchmark scale Ce-LDH, between 25 °C and 1000 °C, is presented in Figure 6. A good resemblance to the TGA curve of Ce-LDH reported by Ye et al., can be noted, i.e., both demonstrate three distinct phases of weight loss at similar temperatures, which confirms that the Ce-LDH structures have similar compositions [38].

The first weight loss (Δm = 12.64%) between 100 °C–350 °C can be attributed to the loss of intramolecular water (1) and dehydroxylation (2), i.e., the loss of interstitial water in the form of OH^−^ ions, that happen in very quick succession [41]. The qualitative representations of the degradation reactions are as follows:

(1) $${\mathrm{Ce}}_{2}{\left(\mathrm{OH}\right)}_{4}\left({\mathrm{SO}}_{4}\right){\mathrm{NO}}_{3}.{\mathrm{xH}}_{2}O\;\left(s\right)\to {\mathrm{Ce}}_{2}{\left(\mathrm{OH}\right)}_{4}\left({\mathrm{SO}}_{4}\right){\mathrm{NO}}_{3}\;\left(s\right)+{\mathrm{xH}}_{2}O\;\left(g\right)$$

(2) $${\mathrm{Ce}}_{2}{\left(\mathrm{OH}\right)}_{4}\left({\mathrm{SO}}_{4}\right){\mathrm{NO}}_{3}\;\left(s\right)\to {\mathrm{Ce}}_{2}O_{2}{\mathrm{SO}}_{4}{\mathrm{NO}}_{3}\;\left(s\right)+2H_{2}O\;\left(g\right)$$

The second, relatively small weight loss (Δm = 2.61%) between 400 °C–550 °C can be attributed to the loss of nitrate ions in the interlayer, originating from the Ce(NO_3_)_3_ 6H_2_O precursor used during synthesis, that leave the structure as NO_x_ gasses [38]. The weight loss is relatively small as the total amount of nitrate groups is negligible due to the preference of LDH materials to intercalate sulfate anions over nitrate anions [64]. The qualitative representation of this degradation reaction is as follows:

(3) $$2{\mathrm{Ce}}_{2}O_{2}{\mathrm{SO}}_{4}{\mathrm{NO}}_{3}\;\left(s\right)+3O_{2}\;\left(g\right)\to \;2{\mathrm{Ce}}_{2}O_{2}{\mathrm{SO}}_{4}\;\left(s\right)+{\mathrm{NO}}_{2}\;\left(g\right)+\mathrm{NO}\;\left(g\right)$$

The final weight loss (Δm = 16.70%) between 700 °C–850 °C is assigned to covalently bound sulfate groups, originating from (NH_4_)_2_S_2_O_8_ during the synthesis of the LDH-structure, that are removed from the structure as SO_x_ gasses [37]. The presence of covalently bound sulfate groups in the Ce-LDH structure was confirmed by Ye et al. through Fourier transform infrared spectroscopy (FT-IR) as the characteristic ν_3_ modes, between 1050 cm^−1^ and 1200 cm^−1^, were observed [38,41]. The removal of these sulfate groups, through calcination at 800°C of the Ce-LDH material, was confirmed through FT-IR analysis by the disappearance of these ν_3_ modes, as illustrated in Figure S2 (see supportive information). Moreover, TGA analyses of Ce_2_O_2_SO_4_, Ce(SO_4_)_2_ and Ce_2_(SO_4_)_3_ in literature all confirm this weight loss between 600 °C and 800 °C [65,66,67]. The qualitative representation of the reaction is as follows:

(4) $$2{\mathrm{Ce}}_{2}O_{2}{\mathrm{SO}}_{4}\left(s\right)+\frac{1}{2}O_{2}\;\left(g\right)\to 4{\mathrm{CeO}}_{2}\;\left(s\right)+{\mathrm{SO}}_{3}\;\left(g\right)\;+{\mathrm{SO}}_{2}\;\left(g\right)$$

#### 3.1.6. Ce-LDH Structural Formula

Based on the TGA analysis, the XPS analysis and the net charge balance of the Ce-LDH material, the structural formula was determined. The in-depth calculations and the mole fractions of water, hydroxyl groups, nitrate anions, sulphate anions and cerium (3+ and 4+) within the Ce-LDH structure can be found in the supportive information (Tables S2 and S3). The structural formula is, finally, found to be Ce^4+^_1_Ce^3+^_1_(OH^−^)_4,15_(SO_4_^2−^)_1,32_(NO_3_^−^)_0,21_.(1.48)H_2_O. This formula is in good agreement with the one reported by Ye et al., which further confirms that the synthesis of the Ce-LDH material was successful [38].

### 3.2. Effect of Support Calcination Temperature

#### 3.2.1. High Temperature X-ray Diffraction

**Figure 7:**
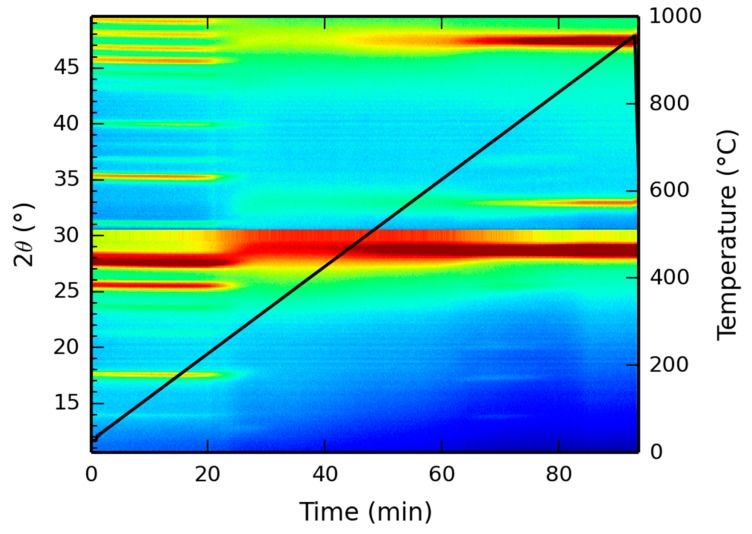
High temperature XRD analysis of the Ce-LDH structure between 0 °C and 1000 °C, heated at 10 °C/min.

X-ray diffraction patterns, as a function of temperature, were recorded up to 1000 °C and the resulting two dimensional mapping is presented in Figure 7. At low temperatures, the XRD pattern corresponds to the XRD pattern as depicted in Figure 3, showing the reflection at 17 °C, indicative of the Ce-LDH structure. However, starting at 220 °C, the dehydroxylation reaction initiates, as experimentally demonstrated by the TGA results in Section 3.1.5. Hence, due to the degradation of the actual hydroxide layers, the characteristic Ce-LDH peaks (e.g., at 17°) disappear. At temperatures above 800 °C, the characteristic peaks of CeO_2_ (JCPDS Card: 043-1002) have fully formed (i.e., at 28.5°, 33° and 47.5°, respectively corresponding to the following Miller indices 111, 200 and 220) [68,69], indicating the complete degradation of Ce-LDH to CeO_2_. This is also confirmed in literature as Ce_2_O_2_SO_4_, Ce(SO_4_)_2_ and Ce_2_(SO_4_)_3_ fully degrade to CeO_2_ at similar temperatures [65,66,67].

#### 3.2.2. Nitrogen Sorption

The BET specific surface areas of CeO_2_ and Ce-LDH, before and after calcination at various temperatures, are presented in Table 3. The pristine Ce-LDH has a BET specific surface area of 11 m²/g, which is significantly lower than that of a traditional hydrotalcite, synthesized through co-precipitation (54 m^2^/g) [70], but matches well with the BET specific surface area reported for monometallic Ce-LDH prepared by Ye et al [38]. This is likely due to the difference in synthesis strategy as, during homogeneous alkalization, OH^−^-ions are generated in situ, resulting in a relatively small supersaturation of the cerium hydroxides, leading to the promotion of crystal growth over nucleation, in turn leading to larger particles. On the other hand, during co-precipitation, the supersaturation of the metal hydroxides is always high due to the increased concentration of OH^−^ ions. Moreover, due to the lower supersaturation, homogeneous alkalization has proven to deliver far more reproducible results for specifically desired metal hydroxide phases [71].

Calcining the material at 600 °C results in the largest BET specific surface area of the Ce-LDH-series (16.17 m^2^/g). On the other hand, the TGA and HT-XRD results, presented in Section 3.1.5 and Section 3.2.1, respectively, indicated that a calcination temperature of 800 °C would lead to complete decomposition of the Ce-LDH structure to CeO_2_, which, in turn, has the highest specific BET surface area of the tested materials. The small difference in BET specific surface area between the CeO_2_ and Ce-LDH-800 can be attributed to the difference of metal oxide synthesis, i.e., degradation of the Ce-LDH structure to CeO_2_ or direct hydrothermal synthesis [25,70].

#### 3.2.3. X-Ray Fluoresence

The weight percentages of sulfur (S) and silicon (Si) in Ce-LDH, Ce-LDH-600 and Ce-LDH-800 were determined through XRF-analysis. Although only semi-quantitative, about 1.51 wt% of sulfur was detected in the Ce-LDH material, which can be attributed to the intercalated sulfate anions of whom the presence was confirmed through XPS in Section 3.1.3. After calcination, the weight fraction of sulfur increases up to 5.38 wt% for Ce-LDH-600, due to the removal of intercalated water and the dehydroxylation reaction, which is also confirmed by the TGA analysis in Section 3.1.5 However, the Ce-LDH-800 material shows near complete removal of sulfur due to the degradation of the Ce_2_O_2_(SO_4_) phase to CeO_2_ during calcination at 800 °C, again, also confirmed through TGA analysis in Section 3.2.1 and FT-IR analysis as illustrated in Figure S2 (see supportive information). Secondly, the XRF analysis confirmed the presence of silicon as impurity, also indicated through XPS in Section 3.1.3, with a low and stochastic abundance between 0.4 wt% and 1.1 wt% for Ce-LDH, Ce-LDH-600 and Ce-LDH-800. 

### 3.3. Characterization of the PdNi-Ce-LDH Catalyst

#### 3.3.1. X-Ray Diffraction

**Figure 8:**
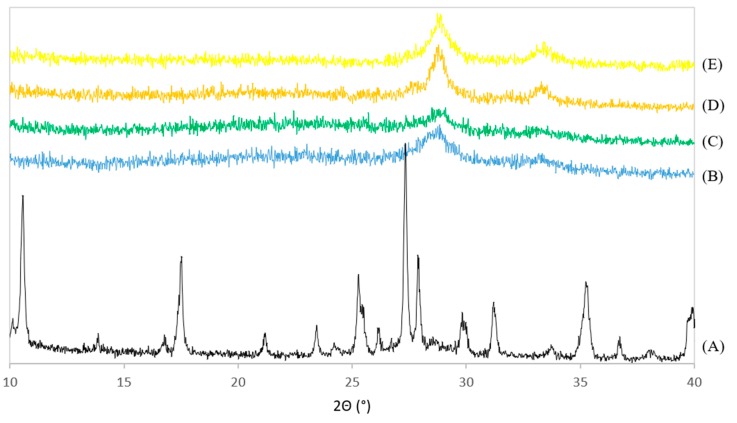
XRD patterns of the uncalcined Ce-LDH support from the scaled-up synthesis (A) and the calcined PdNi-Ce-LDH-200 (B), PdNi-Ce-LDH-400 (C), PdNi-Ce-LDH-600 (D) and PdNi-Ce-LDH-800 (E) catalysts.

The XRD patterns of PdNi-Ce-LDH-200 (B), PdNi-Ce-LDH-400 (C), PdNi-Ce-LDH-600 (D), PdNi-Ce-LDH-800 (E) and the uncalcined Ce-LDH support (A) from the scaled-up synthesis are represented in Figure 8. None of the PdNi-Ce-LDH catalysts exhibit the characteristic reflections of the original Ce-LDH support (e.g., at 10° and 17°), which illustrates the calcination effect. Moreover, all PdNi-Ce-LDH catalysts, regardless of the initial calcination temperature before metal impregnation, illustrate broad reflections at 28.5° and 33°, which is in agreement with the HT-XRD measurements of the Ce-LDH material in Section 3.2.1. Finally, the PdNi-Ce-LDH-600 catalyst shows the highest peak intensity, which is a qualitative indication that this catalyst demonstrates the highest crystallinity. 

#### 3.3.2. Scanning Electron Microscopy and Energy-Dispersive X-Ray Spectroscopy

**Figure 9:**
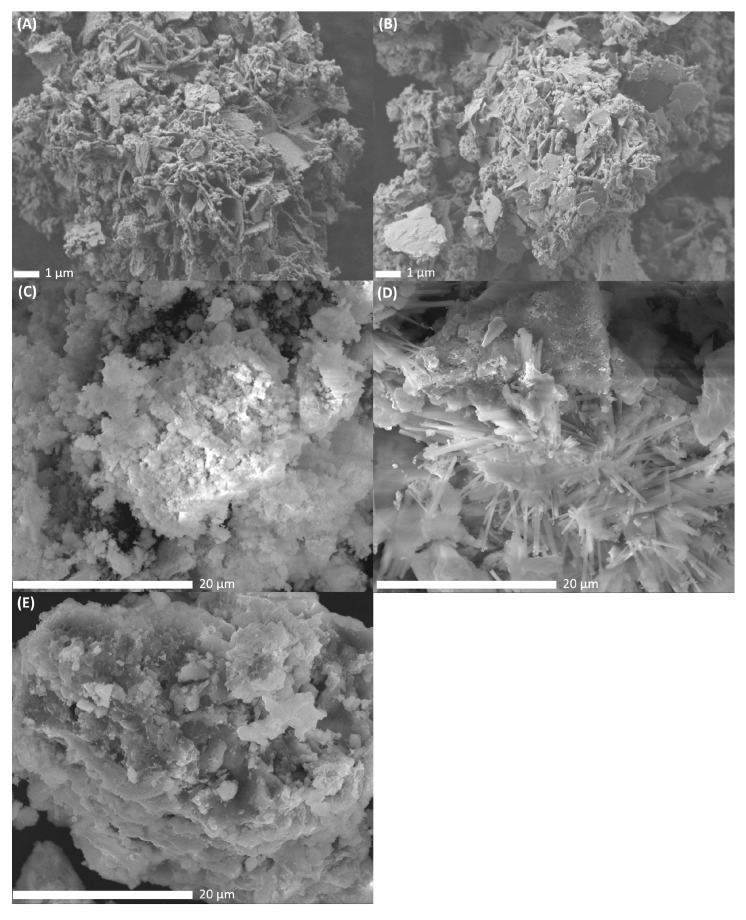
SEM images of the PdNi-Ce-LDH-200 ((**A**) at 7500×), PdNi-Ce-LDH-400 ((**B**) at 7500×), PdNi-Ce-LDH-600 ((**C**) at 7500×), PdNi-Ce-LDH-800 ((**D**) at 7500×) and PdNi-CeO_2_ ((**E**) at 7500×) catalysts.

To study the morphological differences between the catalysts, SEM-images were recorded after metal impregnation, drying and calcination at 500 °C of the catalysts. The resulting SEM-images of PdNi-Ce-LDH-200 (A), PdNi-Ce-LDH-400 (B), PdNi-Ce-LDH-600 (C), PdNi-Ce-LDH-800 (D) and PdNi-CeO_2_ (E) are presented in Figure 9. The PdNi-Ce-LDH-200 and PdNi-Ce-LDH-400 catalysts (Figure 9A,B respectively) exhibit similar morphologies, i.e., clear plate-like structures with intermediate amorphous material, as the effect of the difference in initial calcination temperature of the support itself (i.e., 200 °C and 400 °C in this case) is canceled out by the second calcination step after impregnation (500 °C). The SEM-image of the PdNi-Ce-LDH-600 (Figure 9C) shows lower resolution due to sample charging but the plate-like structures are still present amongst some amorphous material. However, the SEM-images of the PdNi-Ce-LDH-800 catalyst, illustrated in Figure 9D reveal a clear morphological difference as needle-like structures are present throughout the material. Moreover, these structures were not noted by Ye et al. [38] during their SEM-analysis of unloaded Ce-LDH after calcination at 800 °C, nor any other calcination temperature, indicating that they are a consequence of the metal impregnation and second calcination step, rather than the initial calcination of the support. 

As the incipient wetness impregnation uses metal nitrates in aqueous medium, it leads to partial reconstruction of the Ce-LDH phase [25]. Since the TGA results in Section 3.1.5 indicate that only the Ce-LDH-800 support has undergone complete thermal degradation to CeO_2_, the formation of these needle-like structures is most likely related to the reconstruction of the CeO_2_ phase to an LDH structure. Limitation of the LDH reconstruction due to increased calcination temperature has also been reported before for HT [72]. Therefore, it could be hypothesized that this limited reconstruction results in the needle-like morphology. Additionally, SEM-analysis of the PdNi-Ce-LDH-200 and PdNi-Ce-LDH-400 samples and the SEM-analyses of Ye et al. indicate that the morphology of a Ce-LDH phase, but not its composition, is maintained throughout the calcination at 500 °C [38]. On the other hand, next to these needles, big lumps of amorphous material are also present, strongly resembling the morphology of the PdNi-CeO_2_ catalyst, represented in Figure 9D.

Additionally, SEM-EDX analyses were performed to evaluate the metal loading and ratios throughout the Ce-LDH supports. The relative weight and atomic fractions of palladium and nickel on the different Ce-LDH and CeO_2_ supports, as determined by EDX scanning at a magnification of 1000×, are represented in Table 4. Within this Table, a difference in the resulting weight and, subsequently, molar ratios between palladium and nickel can be observed for the different materials. On one hand, the PdNi-CeO_2_ and PdNi-Ce-LDH-800 show near identical Pd:Ni weight ratios of approximately 1:1, which is in accordance to the desired ratio during metal impregnation. On the other hand, the PdNi-Ce-LDH-200, PdNi-Ce-LDH-400 and PdNi-Ce-LDH-600 all demonstrate a slightly different but comparable Pd:Ni weight ratio, in favor of palladium. As the TGA, HT-XRD analyses, in Section 3.1.5 and Section 3.2.1 respectively, and the SEM-images in Figure 9 confirmed that the PdNi-Ce-LDH-800 largely consists of cerium oxide, the difference in Pd:Ni ratio can largely be explained by the local structure of the support itself, i.e., being LDH derived or consisting solely of CeO_2_. This is further confirmed by an EDX spot measurement in the aforementioned needle-like structures of the PdNi-Ce-LDH-800 catalyst, which has a reconstructed Ce-LDH structure due to the memory effect during impregnation, resulting in 67.94 wt% Pd and 32.06 wt% Ni, which is in accordance with the values of the other PdNi-Ce-LDH catalysts. Therefore, it is concluded that within these local measurements, the slight difference in Pd:Ni ratios can be related to the local structure (i.e., being fully converted to CeO_2_ or not). The local deviation from the intended Pd:Ni ratio during metal impregnation, i.e., 1:1 (weight basis), in the PdNi-Ce-LDH catalysts can be explained by very localized agglomeration of nickel. Moreover, within the PdNi-Ce-LDH-400 catalyst, a localized Pd:Ni ratio of 22:78 (weight basis) was found, supporting this hypothesis.

**Figure 10:**
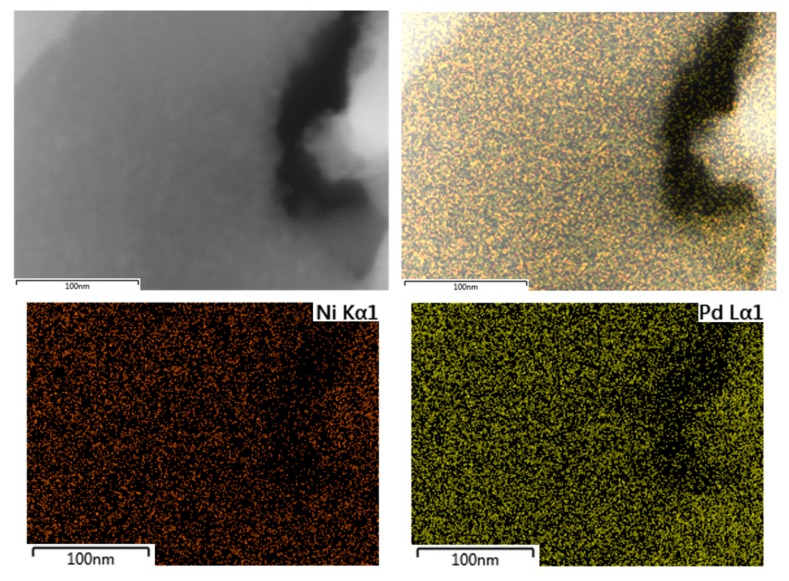
SEM image (**top left**), SEM image with overlay of the palladium and nickel mappings (**top right**), nickel Kα1 EDX mapping (**bottom left**) and palladium Lα1 EDX mapping (**bottom right**) of the PdNi-Ce-LDH-200 catalyst.

Additionally, SEM-EDX mapping of palladium and nickel was performed for the PdNi-Ce-LDH-200 catalyst to study the dispersion. The SEM-image, accompanied by the palladium and nickel EDX mappings and a composite overlay of the mappings on the image, are presented in Figure 10. Based on these images, the distribution of both palladium and nickel is quite homogeneous and no large agglomerates are formed. This indicates that small, well dispersed nanoparticles (i.e., <10 nm in size) are formed, which is, typically, aimed at in the synthesis of supported metal nanoparticles [25].

#### 3.3.3. Catalytic Performance of the Cerium Supported PdNi Catalysts

The BPE conversion after 1 h at 150 °C obtained using the different PdNi-Ce-LDH catalysts are listed in Table 5. The first and most important conclusion, drawn from these results, is that the PdNi-Ce-LDH catalysts, regardless of the initial calcination temperature of the support, outperform the PdNi-CeO_2_ catalyst. Additionally, even the PdNi-Ce-LDH-800 catalysts, hypothesized of only showing partial reconstruction of the Ce-LDH structure during metal impregnation, obtains a BPE conversion (93%) far exceeding that of the PdNi-CeO_2_ material (44%). Moreover, within a control experiment, the unloaded Ce-LDH-600 support material obtained a BPE conversion of 20%, further cementing the high potential of these Ce-LDH derived materials as nanoparticle supports in the hydrogenolysis of lignin.

Secondly, amongst the PdNi-Ce-LDH catalysts, the obtained conversion shows a similar trend, as a function of the calcination temperature, as the BET specific surface area in Section 3.2.2, namely a maximum at 600 °C. However, the difference in surface area between the PdNi-Ce-LDH-200 and PdNi-Ce-LDH-400, due to the difference in calcination temperature of the support (i.e., 200 °C and 400 °C respectively), is canceled out by the second calcination at 500 °C after metal impregnation. Therefore, the trend in conversion cannot be solely related to the surface area of the support and its impact on the metal dispersion. The increase in BPE conversion as a function of the support calcination temperature, is likely linked to the degree of degradation of the Ce-LDH support, which, as illustrated by the TGA analysis in Section 3.1.5, is also a function of the calcination temperature.

A lower initial calcination temperature of the support leads to a less severe collapse of the Ce-LDH structure. However, this leaves more of the structure to collapse under the second calcination, after metal impregnation. Van Vaerenbergh et al., studying Pd nanoparticles on HT, already reported that, during calcination, the collapse of the HT structure around the impregnated metal precursor salts ultimately leads to smaller nanoparticles but also sterically blocks them from the reaction environment, leading to lower catalytic performance [70]. The slight decrease in BPE conversion between the PdNi-Ce-LDH-600 and PdNi-Ce-LDH-800 is likely due to morphological and/or compositional differences of both the support as well as the nanoparticles between the catalysts, illustrated by the SEM-EDX results in Section 3.3.2. Moreover, the XRD results in Section 3.3.1 revealed that the PdNi-Ce-LDH-800 catalyst shows a slightly lower crystallinity, compared to the PdNi-Ce-LDH-600 catalyst. In addition, XPS measurements demonstrated that both the PdNi-Ce-LDH-600 and PdNi-Ce-LDH-800 catalysts display a Ce^3+^/Ce^4+^ ratio of approximately 2/1, which is significantly different from the 1/1 ratio of the uncalcined Ce-LDH and the 92.4% of Ce^4+^ in the CeO_2_ support and could also be related to the optimum in catalytic performance. However, the correlation between the structural, microstructural and morphological properties of the materials and their catalytic performance is very complex and a significant amount of additional research is necessary to fully understand it.

Finally, the results indicate that the synthesized catalysts also show a difference in selectivity as the PdNi-Ce-LDH catalysts show full selectivity towards toluene and phenol while most of the toluene is converted to benzoic acid by the PdNi-CeO_2_ catalyst. As Gao et al. did not report this side reaction for PdAu-CeO_2_ catalysts at 150 °C with water as solvent and formic acid as hydrogen donor, the undesired oxidation mechanism of toluene is likely related to either the nanoparticles (i.e., PdAu versus PdNi) or the solvent and/or hydrogen donor (i.e., water and formic acid versus methanol) [16]. The latter is supported by Deng et al. who reported that Pd-CeO_2_ catalysts are very efficient in the oxidative cleavage of β-O-4 ether linkages under similar conditions, with methanol as a solvent under an oxygen atmosphere, showing certain selectivity towards benzoic acid [18]. Additionally, the oxygen required for the oxidation of toluene to benzoic acid is likely provided by the CeO_2_ as this material is known for its oxidative capabilities due to the liability of the lattice oxygen [22]. Finally, as the PdNi-Ce-LDH-800 catalyst does not demonstrate this oxidative side reaction, it further substantiates the aforementioned proposition that the needle-like structures as depicted in Figure 9 are the result of LDH reconstruction during metal impregnation.

## 4. Conclusions

Monometallic cerium layered double hydroxides (Ce-LDHs) are identified as potentially superior catalytic supports for the reductive cleavage of lignin. This work showed that they can be successfully synthesized, both on a small and larger scale through an HMT-driven homogeneous alkalization route, which was confirmed by XRD, SEM and XPS analyses. The proposed structural formula of the material (Ce^4+^_1_Ce^3+^_1_(OH^−^)_4,15_(SO_4_^2−^)_1,32_(NO_3_^−^)_0,21_.(1,48)H_2_O) was determined through TGA, XPS and iodometric titration. The calcination temperature affects phase changes and the surface area as demonstrated by TGA, HT-XRD and nitrogen sorption results at four calcination temperatures: 200 °C, 400 °C, 600 °C or 800 °C. Three distinctive phases in the thermal degradation of the Ce-LDH material could be identified: the removal of intramolecular water and dehydroxylation, removal of nitrate groups and finally, removal of sulfate groups. Additionally, the Ce-LDH-600 material demonstrated an optimum in BET specific surface area (16.17 m^2^/g) as a function of the calcination temperature. Subsequently, the Ce-LDH materials, calcined at the different aforementioned temperatures, were loaded with 2.5 wt% Pd and 2.5 wt% Ni, calcined at 500 °C, hydrothermally reduced in methanol in presence of hydrogen, tested in the hydrogenolysis of BPE and benchmarked against a CeO_2_ material, loaded similarly. Regardless of the initial calcination temperature of the Ce-LDH support, the PdNi-Ce-LDH catalysts vastly outperformed the PdNi-CeO_2_ catalyst, with the PdNi-Ce-LDH-600 showing the highest BPE conversion (98%). Moreover, while the PdNi-Ce-LDH catalysts showed a full selectivity towards toluene and phenol the PdNi-CeO_2_ catalyst showed an additional side reaction, i.e., oxidizing toluene to benzoic acid. As the PdNi-Ce-LDH catalysts all showed vastly improved BPE conversion and selectivity towards toluene and phenol, compared to the PdNi-CeO_2_ catalyst, the potential of LDH structured materials as a catalyst support in the reductive depolymerization of lignin is significantly substantiated and their performance in the hydrogenolysis of real lignin samples provides an interesting topic for further research. Moreover, this study further proves how the choice of support can significantly influence both catalytic activity and selectivity and how further fine-tuning of the support properties (e.g., steric effects, Ce^3+/^Ce^4+^ ratio, morphology, etc. for this specific support) is necessary to achieve an optimum in catalytic performance.

## Figures and Tables

**Table 1:** Cell parameters of the orthorhombic lattice of the Ce-LDH and scaled-up Ce-LDH.

Material	a (Å)	b (Å)	c (Å)
Ce-LDH	13.79 ± 0.02	7.26 ± 0.01	8.30 ± 0.01
Ce-LDH upscaled	13.51 ± 0.01	7.26± 0.01	8.37 ± 0.01

**Table 2:** Ce^4+^ fraction of the CeO_2_ and Ce-LDH materials as determined by iodometry.

Material	%Ce^4+^ (mol%)
CeO_2_	92.4%
Ce-LDH	46.4%

**Table 3:** BET specific surface area for the CeO_2_ and Ce-LDH materials.

Support Material	BET Specific Surface Area (m²/g)
Ce-LDH	11.00
Ce-LDH-200	12.44
Ce-LDH-400	12.85
Ce-LDH-600	16.17
Ce-LDH-800	13.89
CeO_2_	17.82

**Table 4:** Relative weight and atomic fraction of the loaded palladium and nickel for the different catalysts as determined by localized SEM-EDX measurements at 1000× magnification.

Catalyst	Weight Fraction (%)		Atomic Fraction (%)	
	Pd	Ni	Pd	Ni
PdNi-CeO_2_	48.85	51.15	34.51	65.49
PdNi-Ce-LDH-200	58.75	41.25	44.01	55.99
PdNi-Ce-LDH-400	65.07	34.93	50.69	49.31
PdNi-Ce-LDH-600	62.77	37.23	48.19	51.81
PdNi-Ce-LDH-800	48.35	51.65	34.06	65.94

**Table 5:** BPE conversions for the different PdNi catalysts as determined through HPLC. Reaction conditions: methanol as solvent (20 mL), 750 ppm BPE, catalyst 0.1 g, 600 ppm biphenyl (I.S.), 10 bar H_2_, 150 °C, 1 h, 900 rpm.

Catalyst	Conversion (%)
Ce-LDH 600	20
PdNi/Ce-LDH 200	80
PdNi/Ce-LDH 400	88
PdNi/Ce-LDH 600	98
PdNi/Ce-LDH 800	93
PdNi/CeO_2_^a^	44

^a^ benzoic acid is formed through oxidation of toluene.

## Supplementary Materials

The following supplementary information is available online at https://www.mdpi.com/1996-1944/13/3/691/s1, Figure S1: survey XPS spectrum of the Ce-LDH material between 0 eV and 1000 eV, Figure S2: FT-IR spectra of the PdNi-Ce-600 and PdNi-Ce-LDH-800 catalysts between 600 cm^−1^ and 4000 cm^−1^, Table S1: 2θ values, corresponding d value according to Bragg’s equation, Miller indices ((h,k,l)) and d_hkl_ according to the Miller indices in an orthorhombic lattice for the different reflections in the XRD pattern of the scaled-up Ce-LDH with accompanying calculations, Table S2: Mole fractions of water, nitrate anions and sulphate anions. Calculations are based upon the TGA results and the proposed degradation reactions, Table S3: Calculated mole fractions of intramolecular water, hydroxyl groups, nitrate anions, sulfate anions and cerium (both +III and +IV oxidation states) in the Ce-LDH, based upon the TGA results, XPS analysis and charge balance with accompanying calculations for the proposed structural formula.
