# Supplementary material for: Monometallic Cerium Layered Double Hydroxide Supported Pd-Ni Nanoparticles as High Performance Catalysts for Lignin Hydrogenolysis
Source: Materials (Basel). 2020 Feb 4;13(3):691. doi: 10.3390/ma13030691 (PMC7040693; doi:10.3390/ma13030691)
Supplement: Supplementary file 1 [file materials-13-00691-s001.pdf]

materials

Supplementary Materials: Monometallic Cerium Layered Double Hydroxide supported Pd-Ni Nanoparticles as High Performance Catalysts for Lignin Hydrogenolysis

Tibo De Saegher, Jeroen Lauwaert, Jorku Hanssen, Els Bruneel, Matthias Van Zele, Kevin Van Geem, Klaartje De Buysser and An Verberckmoes *

Miller Indices XRD Spectrum

Table S1. 2θ values, corresponding d value according to Bragg's equation, Miller indices ((h,k,l)) and d_{hkl} according to the Miller indices in an orthorhombic lattice for the different reflections in the XRD pattern of the scaled-up Ce-LDH.

Reflection	2θ (°)	d According to Bragg's Equation (nm)	h	k	l	d_{hkl} According to Calculations (nm)
1	10.56	0.837	0	0	1	0.830
2	17.48	0.507	1	1	1	0.508
3	27.32	0.326	2	2	0	0.321
4	25.26	0.352	2	0	2	0.356
5	29.90	0.299	2	2	1	0.299
6	31.20	0.287	3	1	2	0.284
7	35.34	0.254	2	2	2	0.254
8	39.90	0.226	3	1	3	0.225

For an orthorhombic lattice (a≠b≠c and α=β=γ=0), d_{hkl} can be determined from equation 1. Moreover, using this equation, the Miller indices can be attributed to their respective reflections. These calculations were performed for the scaled-up XRD pattern. Within equation 1, the a, b and c values represent the cell parameters as determined by the TOPAS-academic software package. Through iterative calculations, different Miller indices ((h,k,l)) are tested until the index results in a d value (d_{hkl}) in correspondence with the d value of the specific XRD reflection as calculated using the Bragg equation.

The resulting indices for each reflection in the XRD spectrum of the scaled-up Ce-LDH, are represented in Table S1. The values in table S1 show a good correspondence between the experimental d values (according to Bragg's equation) and the calculated d_{hkl} values (according to the Miller indices, an orthorhombic lattice and the experimental cell parameters). This confirms the proposition of an orthorhombic lattice and allows the Miller indexation of the peaks in the XRD pattern of the Ce-LDH materials.

$$d_{hkl} = \frac{1}{|\frac{h}{a}+\frac{k}{b}+\frac{l}{c}|} = \frac{1}{(\frac{h}{a})^2+(\frac{k}{b})^2+(\frac{l}{c})^2+\frac{2kl}{bc}cos\alpha+\frac{2lh}{ac}cos\beta+\frac{2hk}{ab}cos\gamma} = ((\frac{h}{a})^2+(\frac{k}{b})^2+(\frac{l}{c})^2)^{-1} \quad (1)$$

X-Ray Photoelectron Spectroscopy

The survey spectrum of the Ce-LDH material, from the scaled-up synthesis, between 0 eV and 1000 eV, is presented in Figure S1.

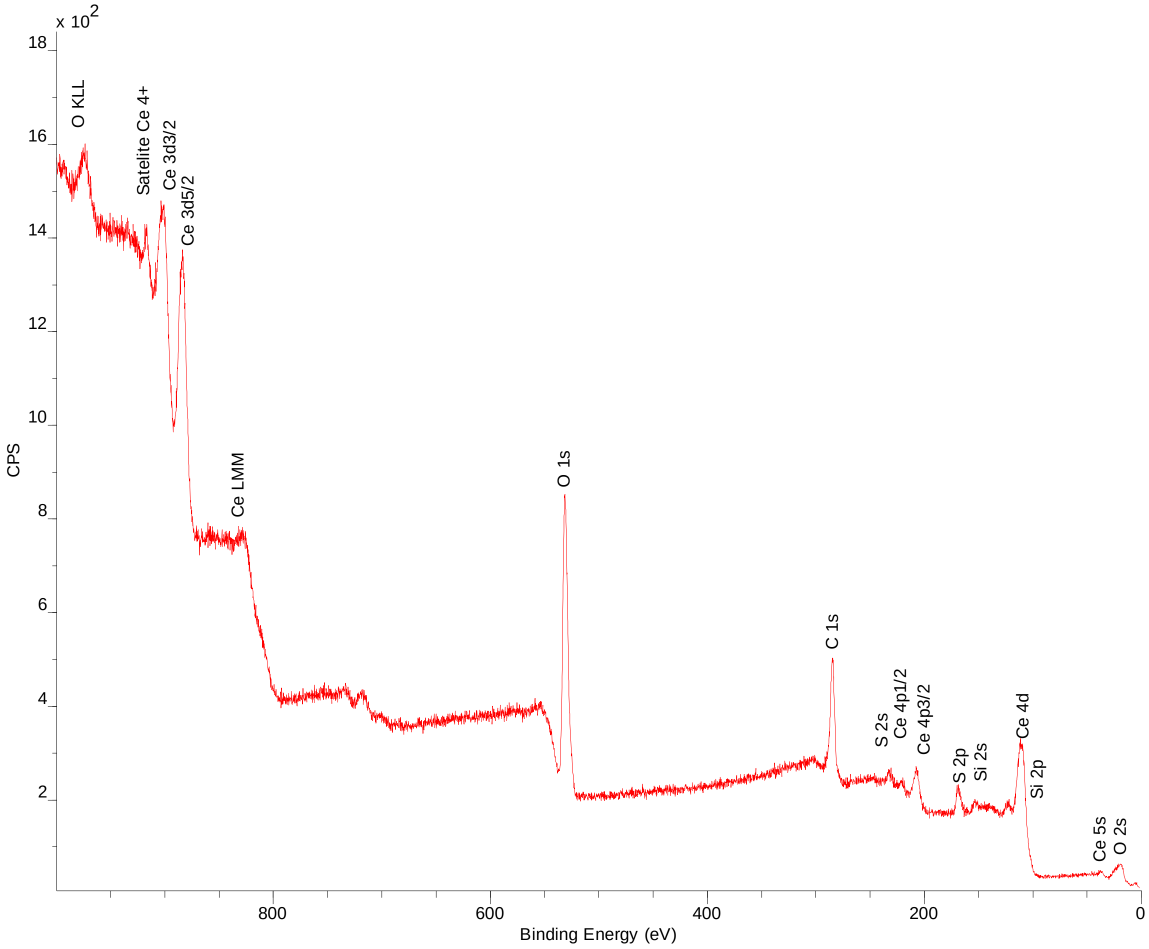

Figure S1. Survey XPS spectrum of the Ce-LDH material from the scaled-up synthesis between 0eV and 1000 eV.

Fourier-Transform Infrared Spectroscopy

The FT-IR spectra of the PdNi-Ce-LDH-600 (A) and PdNi-Ce-LDH-800 (B) catalysts are presented in Figure S2. As indicated by the disappearance of the characteristic ν_3 modes in the PdNi-Ce-LDH-800 spectrum, the sulphate groups are removed through calcination at 800°C of the Ce-LDH material.

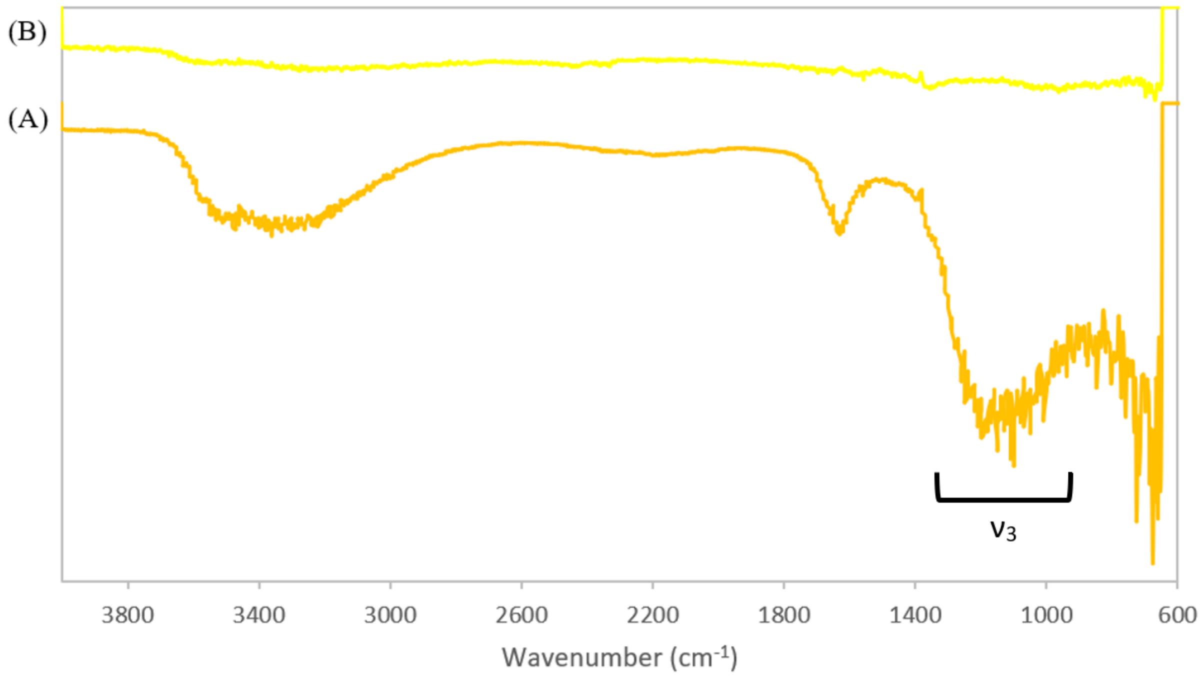

Figure S2. FT-IR spectra of the PdNi-Ce-LDH-600 (A) and PdNi-Ce-LDH-800 (B) catalysts between 600 cm^{-1} and 4000 cm^{-1}.

Determination of the Structural Formula

A Ce-LDH structural formula is proposed based upon the TGA results, XPS analysis and charge balance over the material. Firstly, according to the TGA results, 31.95% of the sample mass is lost between 25°C and 1000°C. Under the hypothesis that the Ce-LDH structure degrades to a pure CeO_2 phase and that each mole of Ce-LDH, containing both Ce^{3+} and Ce^{4+}, results in two moles of CeO_2, the molar mass of the Ce-LDH material can be calculated:

$$MM_{CeO2} = 172.114 \frac{g}{mol} \rightarrow MM_{LDH} = 2 \times \frac{172.114 \frac{g}{mol}}{0.6805} = 505.846 \frac{g}{mol}$$

Additionally, each weight loss step in the TGA-curve, attributed to the removal of specific species, can be used to calculate the mole fraction of water, nitrate anions and sulphate anions in the Ce-LDH material. The results of these calculations are represented in Table S2 and the necessary calculations are elaborated upon.

Table S2. Mole fractions of water, nitrate anions and sulphate anions. Calculations are based upon the TGA results and the proposed degradation reactions.

**Species**	**Mass Loss (%)**	**Mole Fraction of Species in the Ce-LDH Structure**
H_2O	12.64	3.55
NO_3^-	2.61	0.212
SO_4^{2-}	16.7	1.32

- H_2O:

Water is lost both as intramolecular water and hydroxyl groups, the total sample mass loss is 12,64%.

$$\Delta(H_2O) = 12.64\ \% \rightarrow \frac{m_{H2O}}{mol\ LDH} = 0.1264 \times 505.846 = 63.94 \frac{g\ H_2O}{mol\ LDH} = 3.55\ \frac{mol\ H_2O}{mol\ LDH}$$

- NO_3^-:

A small amount of nitrate anions is lost between 400 °C and 550 °C and the total sample mass loss is 2.61%. Within the proposed degradation reactions, these nitrate groups are removed as NO and O_2.

$$\Delta(NO + O_2) = 2.61\,\% \rightarrow \frac{m_{NO3}}{mol\ LDH} = 0.0261 \times 505.846 = 13.2\frac{g\ NO_3}{mol\ LDH} = 0.212\ \frac{mol\ NO_3}{mol\ LDH}$$

- SO_4^{2-}:

The sulphate groups are lost between 600°C and 800°C and the total sample mass loss is 16.7%. Within the proposed degradation reaction, these sulphate groups are removed as SO_3 gas through ambient oxygen (SO_2 + ½ O_2 → SO_3). Therefore, SO_2 is effectively removed from the Ce-LDH structure at these temperatures.

$$\Delta(SO_2) = 16.7\,\% \rightarrow \frac{m_{SO4}}{mol\ LDH} = 0.167 \times 505.846 = 84.476\frac{g\ SO_2}{mol\ LDH} = 1.32\ \frac{mol\ SO_2}{mol\ LDH}$$

The atomic fraction m (Ce^{4+}/(Ce^{3+} + Ce^{4+})) in the Ce-LDH structure was determined to be 50 mol% through XPS analysis and further substantiated through iodometric titration (see Sections 3.1.3 and 3.1.4 respectively). Therefore, the final mole fraction, i.e. the OH^--fraction, can be calculated through the global charge balance of the Ce-LDH material. Again, assuming that two moles of Ce is present per mole of Ce-LDH.

$$(0.5 \times 2) \times 4 + (2 \times 0.5) * 3 = 1.32 \times 2 + 0.212 \times 1 + \frac{n_{OH}}{n_{CeLDH}} \times 1 \rightarrow \frac{n_{OH}}{n_{CeLDH}} = 4.148$$

Based on the proposed degradation reactions in the TGA results, the OH groups in the Ce-LDH structure are removed as water ($Ce_2(OH)_4SO_4 \rightarrow Ce_2O_2SO_4 + 2H_2O$) meaning that 4.148/2= 2.074 mole H_2O are removed during the TGA per mole Ce-LDH which, in turn, means 3.55 − 2.074= 1.48 mole of intramolecular water is present per mole of Ce-LDH. Based on all of these calculations, the mole fractions of the relevant species can be calculated and the results are presented in Table S3.

Table S3. Calculated mole fractions of intramolecular water, hydroxyl groups, nitrate anions, sulphate anions and cerium (both +III and +IV oxidation states) in the Ce-LDH, based upon the TGA results, XPS analysis and charge balance.

Species	Mole Species/Mole Ce-LDH
H_2O	1.48
OH^-	4.15
NO_3^-	0.21
SO_4^{2-}	1.32
Ce^{3+}	1
Ce^{4+}	1

Based on these calculated mole fractions, the following structural formula of the Ce-LDH is proposed:

$$Ce_1^{4+}Ce_1^{3+}(NO_3)_{0.21}^{-}(SO_4)_{1.32}^{2-}(OH)_{4.15}^{-}.\,(1.48)H_2O$$

© 2020 by the authors. Licensee MDPI, Basel, Switzerland. This article is an open access article distributed under the terms and conditions of the Creative Commons Attribution (CC BY) license (http://creativecommons.org/licenses/by/4.0/).
